# Urban forest fragmentation impoverishes native mammalian biodiversity in the tropics

**DOI:** 10.1002/ece3.4632

**Published:** 2018-12-04

**Authors:** Sze Ling Tee, Liza D. Samantha, Norizah Kamarudin, Zubaid Akbar, Alex M. Lechner, Adham Ashton‐Butt, Badrul Azhar

**Affiliations:** ^1^ Department of Forest Management, Faculty of Forestry University Putra Malaysia Serdang Malaysia; ^2^ Faculty of Science and Technology, School of Environmental and Natural Resource Sciences National University of Malaysia Bangi Malaysia; ^3^ School of Environmental and Geographical Sciences University of Nottingham Malaysia Campus Semenyih Malaysia; ^4^ School of Biological Sciences University of Southampton Southampton UK; ^5^ Biodiversity Unit, Institute of Bioscience University Putra Malaysia Serdang Malaysia

**Keywords:** contiguous forest, herbivores, omnivores, species composition, species richness, urban forest patches

## Abstract

Urban expansion has caused major deforestation and forest fragmentation in the tropics. The impacts of habitat fragmentation on biodiversity are understudied in urban forest patches, especially in the tropics and little is known on the conservation value of the patches for maintaining mammalian biodiversity. In this study, camera trapping was used to determine the species composition and species richness of medium‐ and large‐sized mammals in three urban forest patches and a contiguous forest in Peninsular Malaysia. We identified the key vegetation attributes that predicted mammal species richness and occurrence of herbivores and omnivores in urban forest patches. A total number of 19 mammal species from 120 sampling points were recorded. Contiguous forest had the highest number of species compared to the urban forest patches. Sunda Pangolin and Asian Tapir were the only conservation priority species recorded in the urban forest patches and contiguous forest, respectively. Top predators such as Malayan Tiger and Melanistic Leopard were completely absent from the forest patches as well as the contiguous forest. This was reflected by the abundance of wild boars. We found that mammal species richness increased with the number of trees with DBH less than 5 cm, trees with DBH more than 50 cm, and dead standing trees. In the future, the remaining mammal species in the urban forest patches are expected to be locally extinct as connecting the urban forest patches may be infeasible due to land scarcity. Hence, to maintain the ecological integrity of urban forest patches, we recommend that stakeholders take intervention measures such as reintroduction of selected species and restocking of wild populations in the urban forest patches to regenerate the forest ecosystems.

## 1 INTRODUCTION

Forest habitat destruction, fragmentation, and degradation from human activities are the primary drivers of biodiversity loss and negatively affect ecological processes and the provision of ecosystem services (Crooks et al., 2017; Haddad et al., 2015; Lindenmayer & Fischer, 2006). In tropical landscapes, vast swathes of forest are being lost, leaving landscapes composed of smaller patches of forest surrounded by a matrix of human‐modified land cover including the following: agriculture, highways, and human settlements (Fernández & Simonetti, 2013; McKinney, 2008; Pirnat & Hladnik, 2016). Deforestation affects terrestrial biodiversity through the loss of habitat area and the effects of fragmentation (per se) such as patch isolation and increased edge effects (Anderson, Rowcliffe, & Cowlishaw, 2007; Bernard, Fjeldså, & Mohamed, 2009; Melo, Arroyo‐Rodríguez, Fahrig, Martínez‐Ramos, & Tabarelli, 2013).

Besides maintaining ecosystem functions such as pollination and pest control, remnant forest patches in human‐modified landscape are important for providing refugia for wildlife after deforestation (Adila et al., 2017; Ahumada et al., 2011; Brodie et al., 2015; Granados, Crowther, Brodie, & Bernard, 2016; Sasidhran et al., 2016). Remnant patches contribute positively to species persistence as part of a meta‐population according to source‐sink dynamics (Antonini, Martins, Aguiar, & Loyola, 2013; Brodie et al., 2015; Soga, Tamaura, Koike, & Gaston, 2014). Land use conversion forces wildlife to migrate from degraded or hostile areas to suitable habitat within remnant patches or make use of degraded resources within the matrix (Anderson et al., 2007; Gallmetzer & Schulze, 2015; Mukherjee & Sovacool, 2014). However, migration is not always possible for specialist species (McShea et al., 2009). Those species unable to survive in these fragmented landscapes would suffer local extinction (Caughley, 1994).

While there has been a lot of research globally, on the impact of fragmentation (e.g., Anderson et al., 2007; Sodhi et al., 2010; Seto, Güneralp, & Hutyra, 2012; Newbold et al., 2015), yet the impacts of fragmentation due to urbanization in tropical forest landscapes are less studied (e.g., Azhar, Lindenmayer, Wood, Fischer, & Zakaria, 2014; Sasidhran et al., 2016; Adila et al., 2017). Densely populated urban matrix is likely to be more inhospitable to forest species compared to less populated agricultural matrix (Azhar et al., 2015; Poessel, Breck, & Gese, 2016). Medium‐ to large‐sized mammals are classified as indicator species which are susceptible to ecosystem changes (Azlan & Sharma, 2006; Tobler, Carrillo‐Percastegui, Leite Pitman, Mares, & Powell, 2008). The mammals can be used as indicator of ecosystem health and perform important ecosystem functions such as seed dispersal, pest control, and pollination. Hence, quantifying their diversity in the urban forest patches is pivotal for developing conservation strategies (Granados et al., 2016).

**Figure 1:**
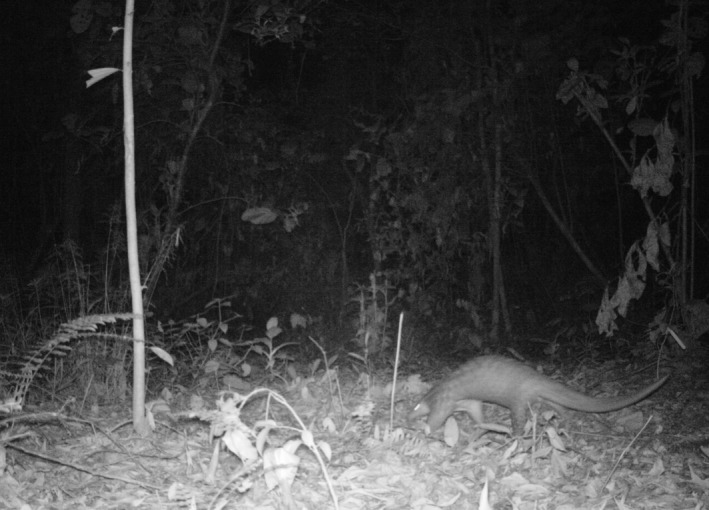
Camera trapping technology was used to study elusive and rare mammals such as Sunda Pangolin (*Manis javanica*) in urban forest fragments

In this study, we examined the effects of habitat disturbance within urban forest fragments on native mammals and their ecosystem functioning using nonintrusive motion‐triggered camera traps (Figure 1). We assessed the following research questions: (a) Do urban forest patches sustain mammal diversity? We predicted the urban forest remnants will favor generalist species as they have a wider ecological acceptance range. (b) What are the key environmental drivers of mammal and functional diversity in these urban forest patches? We predicted that patches that contained more large trees would have greater mammal species richness. (c) Are there any differences of the mammal species composition between each forest patch? We predicted larger forest patches would have more diverse mammal species composition compared to smaller forest patches as more resources are available in the former. The findings of this study are crucial to justify the conservation of forest patches within tropical urban landscapes.

## 2 METHODS

### 2.1 Study areas

**Figure 2:**
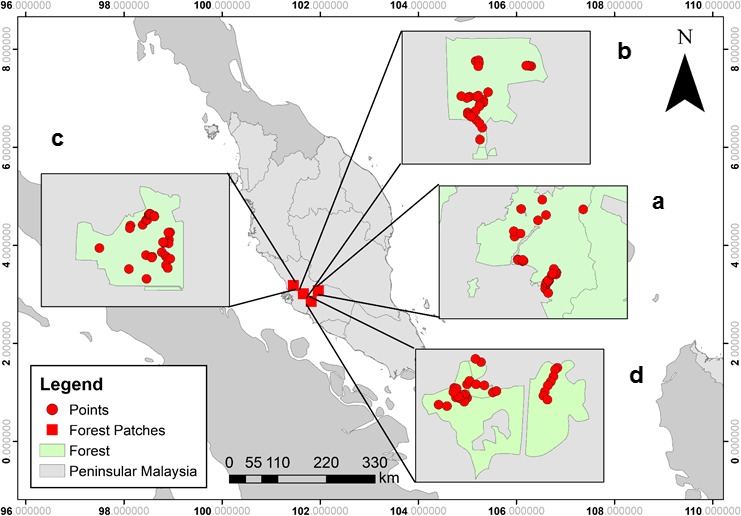
Map of Peninsular Malaysia, where the red squares represent the four study site locations in the state of Selangor: (a) contiguous forest, (b) Patch 1, (c) Patch 2, (d) Patch 3. The red points represent the approximate location of camera trap at each site

This study was conducted in the state of Selangor, Malaysia across three urban forest patches and a contiguous forest within or near Greater Kuala Lumpur, a region including around eight million people (Department of Statistics Malaysia Official Portal, 2017; Figure 2). The three urban forest patches are found deep within a matrix of high‐density urbanization dissected by motorways. These locations were selected as they share similar climatic, edaphic, and topographic conditions. The forests are classified as secondary lowland and hill dipterocarp forest, and have been logged, though more than 30–40 years ago.

The first study location was the Sungai Lalang Forest Reserve, (contiguous forest; 3°9′35.71″N, 102°0′16.51″E), a part of the Langat Basin Reserve, located in the Southern part of Selangor and Northern part of Negeri Sembilan state (Figure 2a). It is characterized by lowland and hill dipterocarp forest with an altitude of 50 m to 800 m above sea level. The reserve is a contiguous forest with some small roads. It is part of a contiguous network of reserves and forested private land which hold the majority of forest in Peninsular Malaysia. The estimated area for SLFR is about 50,000 ha with 82 ha of virgin jungle reserve (VJR) within it (Laidlaw, 2000).

The second location was the Ayer Hitam Forest Reserve (Patch 1; 3°1′12.52″N, 101°38′46.76″E) and is one of the few remaining isolated secondary lowland dipterocarp forests with an altitude of 15 m to 233 m above sea level. It is located in Puchong, Selangor. It has decreased in area from 4,271 ha in 1906 to its current size of 1,176 ha due to rapid urbanization and industrialization in the area (Figure 2b). It is approximately 35 km from the contiguous forest.

The third location was the Bukit Cerakah Forest Reserve (Patch 2). It is 817 ha of isolated forest located near the center of Greater Kuala Lumpur (3°6′34.43″N, 101°30′10.17″E). Patch 2 has an altitude of up to 200 m above sea level and is surrounded by housing which is rapidly increasing in density (Figure 2c). The distance between Patch 2 and contiguous forest is approximately 45 km.

The final location, the Bangi Forest Reserve (Patch 3; 2°54′50.68″N, 101°46′1.18″E), is the smallest forest fragment included in the study. It has an altitude of 40 m to 110 m above sea level and is located in Hulu Langat, Selangor. The forest is isolated and under the pressure from urban and industrial development. Of the 138 ha of gazetted forest reserve, 81 ha is an ecological research area (Figure 2d). This isolated remnant is embedded in a matrix of highways, the Langat River, residential areas, rubber plantations, and oil palm plantations. The estimated distance between Patch 3 and contiguous forest is approximately 20 km.

Patch 1, Patch 2, and Patch 3 were the only sizeable forest patches (>100 ha) that are present in the region. Except Patch 3, all study sites were inhabited by local indigenous people who have lived at the edge of forests. They were allowed by law to access the forest reserves, harvest natural resources, and practise subsistence hunting.

### 2.2 Sampling design

Cameras were randomly deployed at 30 sampling points located within each of the four study sites with a minimum distance of 200 m apart from one another particularly in small patches (Figure 2). Each camera deployment point was chosen based on the presence of visible animal trails, footprints, scents, activity areas (e.g., big wallows left by Eurasian wild pig) and tree marks by wildlife (e.g., scratching marks of sun bear on tree trunks; Sasidhran et al., 2016) or next to streams. Baits were not used in this study to avoid any specific preference or bias, with the aim of surveying medium‐ (>1 kg) and large‐sized nonflying mammal species (Tobler et al., 2008).

### 2.3 Camera trapping

Camera trapping surveys were conducted between October 2016 and October 2017. Thirty cameras (Bushnell Trophy Cam and Bushnell Trophy Cam HD) were used in the survey for 24 hr per day for a total of two weeks at each location to obtain adequate data for the analysis (Nichols & Karanth, 2002). These cameras used infrared camera sensors triggered by heat and motion set at 1‐s interval between exposures. The cameras were installed on suitable trees with a height of 30–50 cm above the ground at optimum angles for overlooking the animal trails without the camera view being blocked by any objects. We identified mammal species based on several identification guides (Francis & Barrett, 2008; Medway, 1978). The mammal species were then further categorized according to feeding guild using the guides. Image of rain, wind, overexposure, blurred, insects, snakes, small rodents, feral or free‐ranging dogs, domestic cats, and human activities were excluded in this study.

### 2.4 Habitat variables

In order to investigate the influence of habitat quality on mammal species richness, 20 m × 20 m vegetation plots were established for each camera deployment location. Thirteen variables were counted, measured, or determined as follows: (a) the number of trees with DBH less than 5 cm; (b) the number of trees with DBH between 5 cm to 30 cm; (c) the number of trees with DBH between 30 cm to 50 cm; (d) the number of trees with DBH greater than 50 cm; (e) the number of palm; (f) the number of trees with liana; (g) the number of shrubs, that is, woody plants with multiple stems and stand less than 6 m; (h) the number of dead standing trees; (i) the number of dead fallen trees; (j) canopy cover; (k) altitude; (l) proximity to human settlement, measured using Google Earth Pro; and (m) habitat type (e.g., contiguous forest, Patch 1, Patch 2, and Patch 3). We followed Sasidhran et al. (2016) and Adila et al. (2017) to justify the selection of the variables.

### 2.5 Statistical analysis

Similarity Percentage (SIMPER) analysis was performed to identify which species contributing most to the differences in mammal assemblages. Bray Curtis Similarity with a 100% cutoff was used to determine the composition of species at each of the four study locations. Analysis of Similarity (ANOSIM) was performed to identify the differences in species composition between each of the study locations, where *R*‐values closer to 1 indicate a higher degree of separation. All analyses were conducted using Primer version 6 software.

To identify the relationship between mammal species richness and feeding guild with the habitat variables, we used Generalized Linear Models (GLMs). The feeding guild was used as a response variable and was grouped into herbivore, omnivore, and carnivore. First, correlation tests were conducted to determine multicollinearity among all the 13 explanatory variables. Explanatory variables which showed strong collinearity (|*r*| > 0.7) were dropped from the subsequent analysis (Dormann et al., 2013). We regressed the number of mammal species, the number of herbivore detections, and the number of omnivore detections recorded in each forest site against the remaining explanatory variables. Regression was not performed on carnivores due to the small number of detections. A Log‐link function was used in the model, and *p*‐value for significant explanatory variables was reported. We tested all possible regression models and selected the “best” model based on the largest *R*‐squared values with the smallest number of covariates. Large *R*‐squared values reflect the reliability of fitted values and a small number of covariates represent a more parsimonious solution. These analyses were performed using GenStat 12th version (VSN International, Hemed Hempstead, UK).

The spatial autocorrelation in residuals was examined by calculating Global Moran's Index in the ArcGIS™ version 10.1 (ESRI). We used the *p*‐value to reject or accept the null hypothesis which states that the analyzed attribute is randomly distributed among the features in the study area (Mitchell, 2005). Inverse distance (nearby neighboring features have a larger influence on the computations for a target feature than features that are far away) was used to compute Global Moran's Index. We used Euclidean distance (the straight‐line distance between two points) as the distance method.

## 3 RESULTS

### 3.1 Mammal species richness

**Figure 3:**
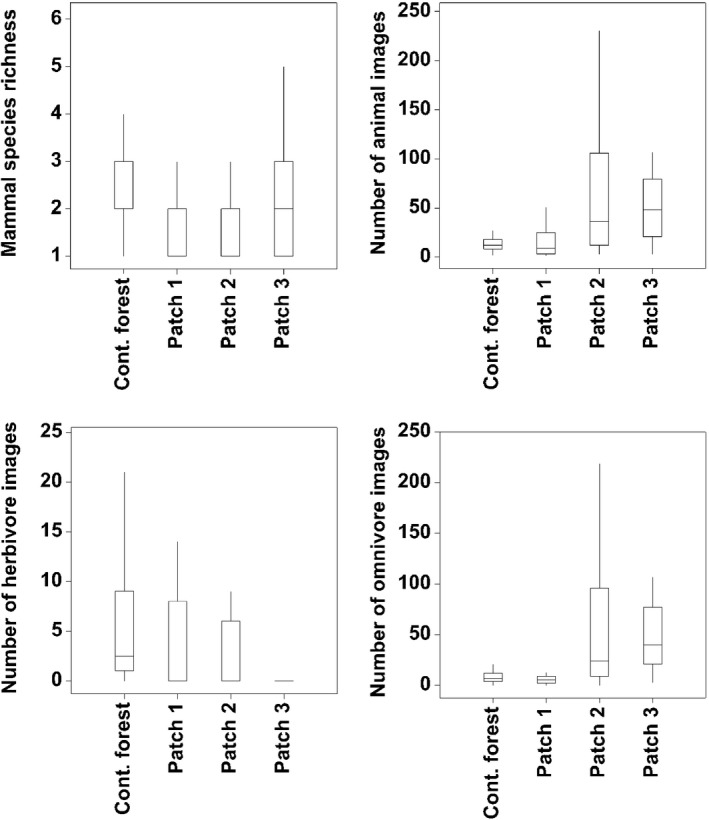
Box‐plots of mammal species richness, the number of animal photographs, the number of herbivore photographs and the number of omnivore photographs that were captured at each study site

The total field effort comprised 120 camera traps, over a cumulative period of 1,680 days, recorded a total of 5,494 photographs which included detection for a number of IUCN listed species. A total of 19 species from 12 families were recorded from the four study areas. The smallest forest patch (i.e., Patch 3) had the highest detections at 2,109, followed by Patch 2 at 2,087, then contiguous forest with 690 photographs and lastly Patch 1 with 608 photographs (Figure 3). The majority of the detection comprised omnivores (82.07%, *n* = 4,509), followed by herbivores (16.67%, *n* = 916) and lastly carnivores (1.26%, *n* = 69; Figure 3).

**Figure 4:**
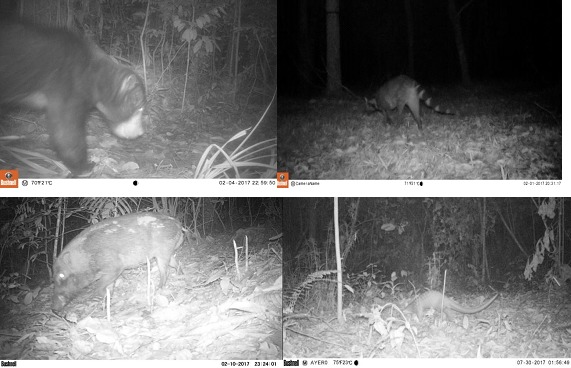
Animals captured by camera traps: *Helarctos malayanus* in contiguous forest (top left); *Viverra megaspila* in Patch 1 (top right); (b); *Sus scrofa* in Patch 2 (bottom left); and *Manis javanica* in Patch 3 (bottom right)

The most surprising result was the presence of listed species in the urban forest patches, especially the critically endangered, *Manis javanica* (Sunda Pangolin) in Patch 3 and Patch 1 (Table 1). In addition, two of the other species recorded, *Tapirus indicus* (Malayan Tapir) and *Hylobates lar* (White‐handed Gibbon), are classified as endangered. *Macaca nemestrina* (Pig‐tailed Macaque) and *Herlactos malayanus* (Malayan Sun Bear) were also detected and both are listed as vulnerable. *Hemigalus derbyanus* (Banded Civet), *Viverra megaspila* (Large Indian Civet), and *Presbytis siamensis* (White‐thighed Langur) were recorded and are classified as near threatened (Figure 4). The rest of the species recorded are classified as least concern species. There were four common species which could be found at all study sites: *Macaca nemestrina*,* Macaca fascicularis*,* Sus scrofa,* and *Tragulus kanchil*. In addition, our camera captured photographs of several domestic dogs in the urban forest patches (55, four, and 85 photographs from Patch 1, Patch 2, and Patch 3, respectively) as well as contiguous forest (seven photographs). However, these photographs were collected from less than 16% of the sampling points. The dogs were mostly detected at nine and five sampling points from Patch 1 and Patch 3, respectively. Only two sampling points each from Patch 2 and contiguous forest had the detection of dogs.

**Table 1:** Species list with the number of photographs captured; feeding guild; and IUCN status for each study site

Species	Family	IUCN status	Feeding guild	Contiguous forest	Patch 1	Patch 2	Patch 3	# of photographs
*Macaca nemestrina* (Pig‐tailed Macaque)	Cercopithecidae	VU	Omnivore	170	275	677	515	1637
*Macaca fascicularis* (Long‐tailed Macaque)	Cercopithecidae	LC	Omnivore	33	35	51	276	395
*Sus scrofa* (Eurasian Wild Pig)	Suidae	LC	Omnivore	161	44	918	1,198	2,321
*Tragulus kanchil* (Lesser Mousedeer)	Tragulidae	LC	Herbivore	65	233	354	58	710
*Paradoxurus hermaphrodites* (Common Palm Civet)	Viverridae	LC	Omnivore	2	—	45	—	47
*Hemigalus derbyanus* (Banded Civet)	Viverridae	NT	Carnivore	9	—	—	—	9
*Paguma larvata* (Masked Palm Civet)	Viverridae	LC	Omnivore	6	—	—	—	6
*Hystrix brachyura* (Malayan Procupine)	Hystricidae	LC	Omnivore	24	—	—	49	73
*Muntiacus muntjak* (Barking Deer)	Cervidae	LC	Herbivore	175	—	—	—	175
*Prionailurus bengalensis* (Leopard Cat)	Felidae	LC	Carnivore	39	—	—	—	39
*Helarctos malayanus* (Sun Bear)	Ursidae	VU	Omnivore	4	—	—	—	4
*Tapirus indicus* (Malayan Tapir)	Tapiridae	EN	Herbivore	1	—	24	—	25
*Hylobates lar* (White‐handed Gibbon)	Hylobatidae	EN	Omnivore	1	—	—	—	1
*Viverra megaspila* (Large Indian Civet)	Viverridae	NT	Carnivore	—	12	—	—	12
*Arctogalidia trivirgata* (Small‐toothed Palm Civet)	Viverridae	LC	Omnivore	—	3	18	—	21
*Viverricula indica* (Small Indian Civet)	Viverridae	LC	Omnivore	—	3	—	—	3
*Manis javanica* (Sunda Pangolin)	Manidae	CE	Insectivore	—	3	—	6	9
*Martes flavigula* (Yellow‐throated Marten)	Mustelidae	LC	Omnivore	—	—	—	1	1
*Presbytis siamensis* (White‐thighed Langur)	Cercopithecidae	NT	Herbivore	—	—	—	6	6

CE: critically endangered; EN: endangered; LC: least concern; NT: near threatened; VU: vulnerable.

### 3.2 Species composition at each study area

Mammal species composition analyzed with SIMPER showed that species composition varied between study sites (Table 2). In the contiguous forest *S. scrofa*,* Muntiacus muntjak*,* M. fascicularis,* and *T. kanchil* represented 93% of the species composition. The remaining 7% were composed of *M. nemstrina*,* Hystrix brachyura*,* Paguma larvata*,* H. derbyanus,* and *H. malayanus*. Patch 1 was the least diverse with the majority of the composition made up of two species, *M. nemestrina* and *T. kanchil,* representing 79.84% and 18.70% of the species composition, respectively. Two other species were also found on the site, representing 1% of the mammal composition, *S. scrofa* and *M. fascicularis*. In Patch 2, 91% of species composition was comprised of *S. scrofa* (63.42%) and *M. nemestrina* (27.75%) and *T. kanchil*;* T. indicus; M. fascicularis*; and *Arctogalidia trivirgata* made up approximately 9% of the species composition. In Patch 3, more than 90% of species composition was contributed by *S. scrofa* (54.70%) and *M. nemestrina* (39.76%). The remaining 5% were made up by *M. fascicularis* (2.65%), *H. brachyura* (2.31%), *T. kanchil* (0.51%), and *M. javanica* (0.07%).

**Table 2:** Mammal species composition at each study site quantified using SIMPER analysis with a 100% cutoff

Habitat	Species	Average abundance	Contribution (%)	Cumulative contribution (%)
Contiguous forest (50,000 ha)	*Sus scrofa*	1.81	51.17	51.17
	*Muntiacus muntjak*	1.67	28.60	79.77
	*Macaca fascicularis*	0.56	7.55	87.33
	*Tragulus kanchil*	0.68	6.09	93.41
	*Macaca nemestrina*	1.02	4.84	98.25
	*Hystrix brachyuran*	0.38	1.57	99.82
	*Paguma larvata*	0.12	0.09	99.91
	*Hemigalus derbyanus*	0.14	0.05	99.96
	*Helarctos malayanus*	0.09	0.04	100
Patch 1 (4,271 ha)	*Macaca nemestrina*	2.25	79.84	79.84
	*Tragulus kanchil*	1.56	18.70	98.55
	*Sus scrofa*	0.42	0.88	99.42
	*Macaca fascicularis*	0.31	0.58	100
Patch 2 (817 ha)	*Sus scrofa*	4.05	63.42	63.42
	*Macaca nemestrina*	3.04	27.75	91.17
	*Tragulus kanchil*	1.52	6.98	98.15
	*Tapirus indicus*	0.32	1.32	99.47
	*Macaca fascicularis*	0.37	0.43	99.90
	*Arctogalidia trivirgata*	0.19	0.10	100
Patch 3 (183 ha)	*Sus scrofa*	4.89	54.7	54.7
	*Macaca nemestrina*	3.18	39.76	94.46
	*Macaca fascicularis*	1.35	2.65	97.11
	*Hystrix brachyuran*	0.63	2.31	99.42
	*Tragulus kanchil*	0.46	0.51	99.93
	*Manis javanica*	0.12	0.07	100

### 3.3 Species composition similarity between study sites

The pair‐wise test (Table 3) indicated species composition between contiguous forest and Patch 1, Patch 2, and Patch 3 was significantly different with *R*‐values of 0.43; 0.21; and 0.30, respectively. Pair‐wise test between Patch 1 with Patch 2 and Patch 1 with Patch 3 was also significantly different with low *R*‐values of 0.26 and 0.33, respectively. Only the pairwise comparison between Patch 2 and Patch 3 had a nonsignificant correlation and the lowest *R*‐value at 0.03.

**Table 3:** Differences in species composition between each of the study site assessed with an ANOSIM

Groups	*R* statistic	*p*‐Value
Cont. forest; Patch 1	0.434	0.010
Cont. forest; Patch 2	0.214	0.010
Cont. forest; Patch 3	0.304	0.010
Patch 1; Patch 2	0.257	0.010
Patch 1; Patch 3	0.327	0.010
Patch 2; Patch 3	0.026	0.109

### 3.4 Habitat variables and mammal species richness

**Figure 5:**
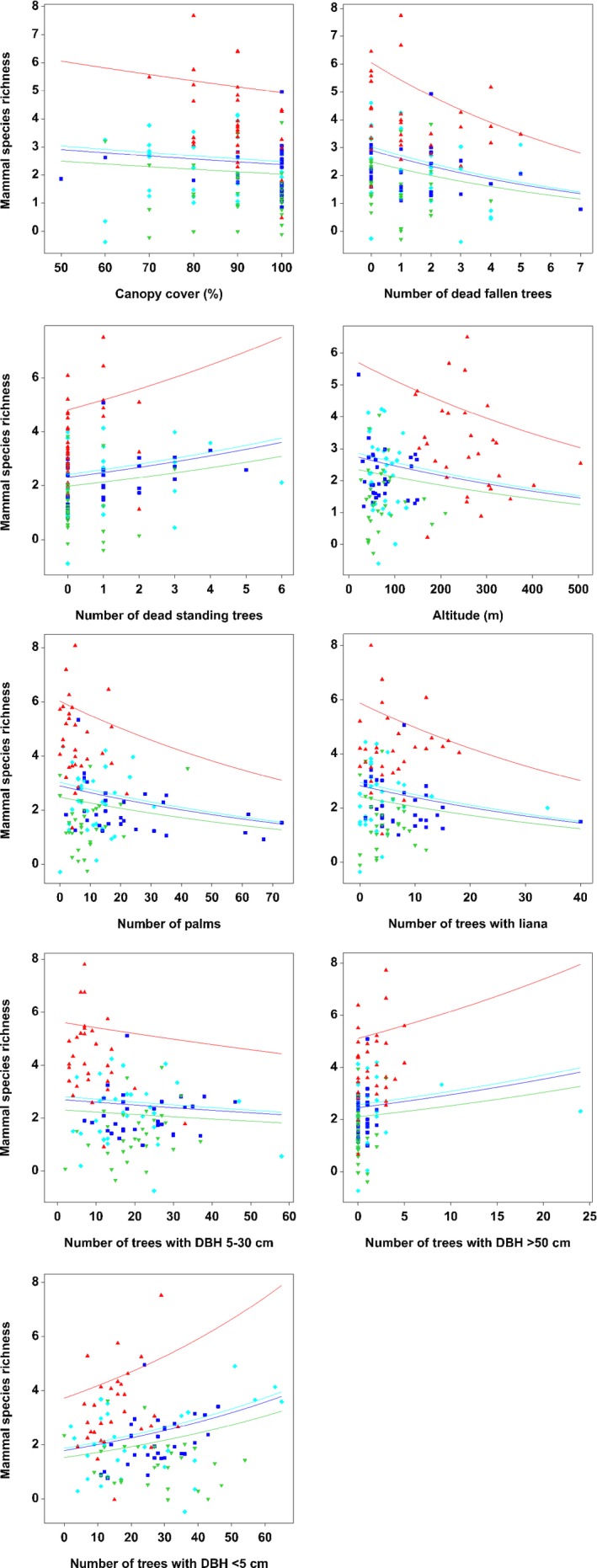
Scatter plots with regression lines showing the relationship between the mammal species richness with habitat quality attributes for all four study sites. The red line represents contiguous forest, green line represents Patch 1, dark blue line represents Patch 2, and light blue line represents Patch 3. The fitted relationship is plotted on the original scale, but the option to use the scale of the linear predictor was selected to check for potential nonlinearity in the response

The number of trees with DBH between 30 cm and 50 cm was removed from modeling process as it was strongly correlated (*r* = −0.778) with the number of trees with DBH greater than 50 cm (Supporting Information Appendix S1). The final model comprised ten explanatory variables (*R* ^2^ = 45.85%) from the original 13 variables (Table 4). These variables were habitat type, altitude, the number of palm, the number of trees with liana, canopy cover, the number of dead fallen trees, the number of dead standing trees, the number of trees with DBH less than 5 cm, the number of trees with DBH between 5 cm and 30 cm, and the number of trees with DBH more than 50 cm. Our main result indicated that the mammal species richness was significantly lower (*p* < 0.001) in all urban forest patches compared to contiguous forest (Table 4). In summary, species richness decreased with canopy cover (*p* < 0.001), the number of dead fallen trees (*p* < 0.001), altitude (*p* < 0.001), the number of trees with lianas (*p* < 0.001), the number of palms (*p* < 0.001), and the number of trees with DBH between 5 cm and 30 cm (*p* = 0.002; Figure 5). Species richness increased with number of dead standing trees (*p* < 0.001), the number of trees with DBH greater than 50 cm (*p* < 0.001), and the number of trees with DBH less than 5 cm (*p* < 0.001; Figure 5). We did not detect a significant effect of the number of shrubs and proximity to human settlement on mammal species richness.

**Table 4:** GLMs of mammal species richness and feeding guild response to explanatory variables

Independent variables	Dependent variables					
	Mammal species richness		Herbivore occurrence		Omnivore occurrence	
	Slope	Wald statistics	Slope	Wald statistics	Slope	Wald statistics
The number of trees with DBH <5 cm	0.012	350.57	0.037	132.99	0.035	798.50
The number of dead fallen trees	−0.110	215.55	−0.193	37.30	−0.078	47.50
The number of trees with liana	−0.017	104.30	−0.065	62.22	0.017	51.00
Altitude (m)	−0.001	17.47	−0.009	68.13	−0.010	285.70
The number of trees with DBH >50 cm	0.018	15.52	0.1160	37.14		
The number of trees with DBH 5–30 cm	−0.004	9.89	−0.023	25.28		
The number of shrubs			−0.005	14.33	−0.007	96.10
Canopy cover (%)	−0.004	20.48			−0.020	235.80
The number of dead standing trees	0.074	64.42			0.148	151.60
The number of trees with DBH 30–50 cm					0.006	9.30
The number of palms	−0.009	96.31			−0.004	9.40
Proximity to human settlement			0.531	34.82		
Habitat type		167.62		194.29		753.90
Patch 1	−0.888		−1.426		−2.33	
Patch 2	−0.734		−0.790		−1.013	
Patch 3	−0.690		−2.561		−0.747	

### 3.5 Habitat variables relationship with herbivore occurrence

**Figure 6:**
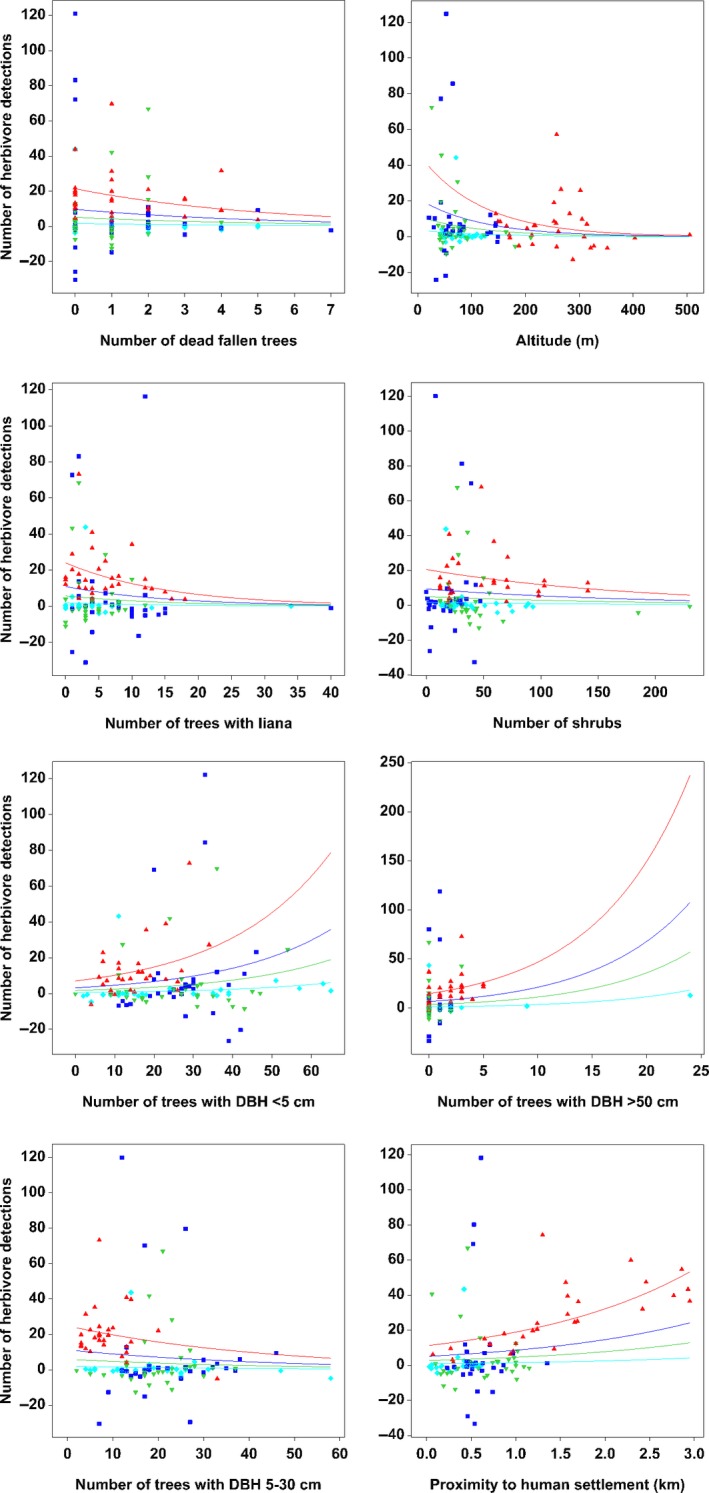
Scatter plots with regression lines showing the relationship between the herbivore detections with habitat variables for all study sites. The red line represents contiguous forest, green line represents Patch 1, dark blue line represents Patch 2, and light blue line represents Patch 3. The fitted relationship is plotted on the original scale, but the option to use the scale of the linear predictor was selected to check for potential nonlinearity in the response

The number of trees with DBH 30 cm to 50 cm was excluded from modeling process as it was strongly correlated (*r* = −0.728) with the number of trees with DBH greater than 50 cm (Supporting Information Appendix S2). The final model comprised nine explanatory variables (*R* ^2^ = 22.04%; Table 4). Our results revealed that the number of herbivore detection was significantly lower (*p* < 0.001) in all urban forest patches compared to contiguous forest (Table 4). Overall, the number of herbivore detection increased significantly with the number of trees with DBH less than 5 cm (*p* < 0.001), the number of trees with DBH greater than 50 cm (*p* < 0.001) and the proximity to human settlement (*p* < 0.001), but decreased with the number of dead fallen trees (*p* < 0.001), the number of trees with liana (*p* < 0.001), altitude (*p* < 0.001), the number of trees with DBH between 5 cm to 30 cm (*p* < 0.001), and the number of shrubs (*p* < 0.001; Figure 6). While the canopy cover, the number of dead standing trees and the number of palm did not have significant effect on the number of herbivore detection.

### 3.6 Habitat variables relationship with omnivore occurrence

**Figure 7:**
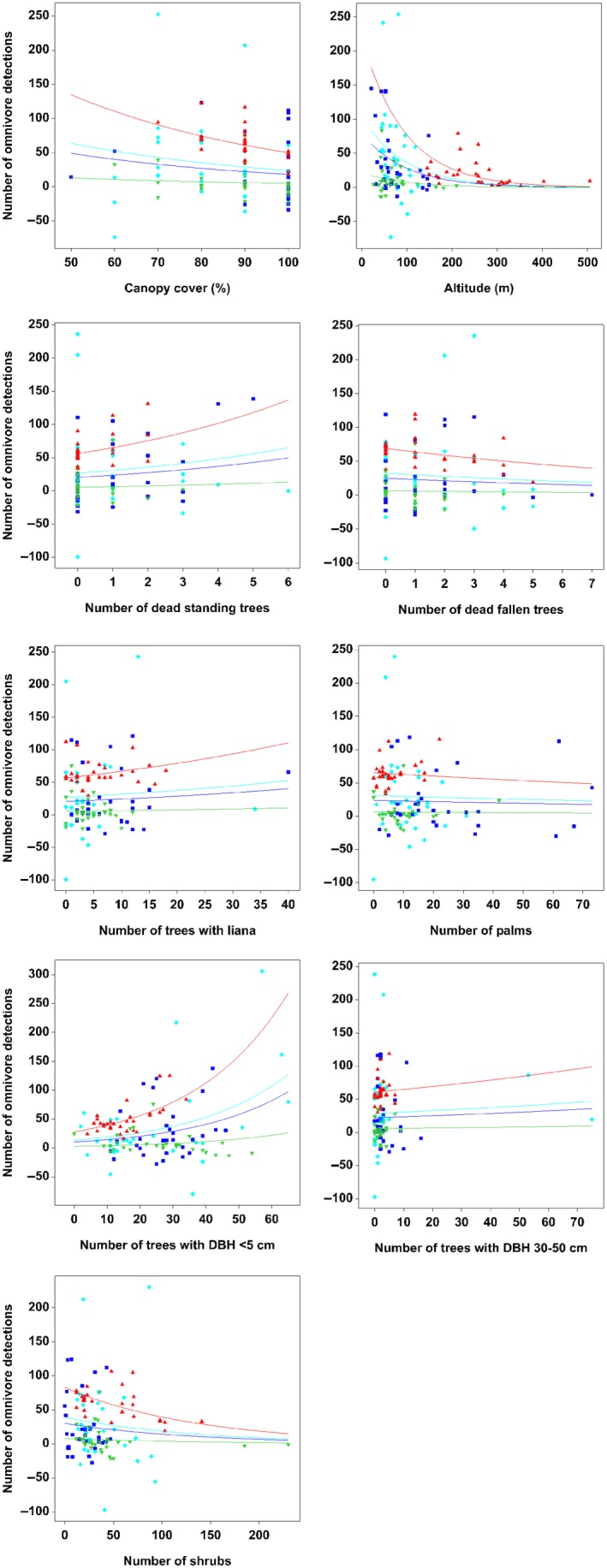
Scatter plots with regression lines showing the relationship between the omnivore detections with habitat variables for all study sites. The red line represents contiguous forest, green line represents Patch 1, dark blue line represents Patch 2, and light blue line represents Patch 3. The fitted relationship is plotted on the original scale, but the option to use the scale of the linear predictor was selected to check for potential nonlinearity in the response

The number of trees with DBH greater than 50 cm was dropped from modeling process because it was strongly correlated (*r* = −0.853) with the number of trees with DBH between 30 cm and 50 cm (Supporting Information Appendix S3). The final model encompassed ten explanatory variables (*R* ^2^ = 54.63%; Table 4). We found that the number of omnivore detection was significantly lower (*p* < 0.001) in all urban forest patches compared to contiguous forest (Table 4). The number of omnivore detection decreased significantly with altitude (*p* < 0.001), canopy cover (*p* < 0.001), the number of shrubs (*p* < 0.001), the number of dead fallen trees (*p* < 0.001), and the number of palm (*p* = 0.002; Figure 7). However, the number of omnivore detection increased with the number of trees with DBH less than 5 cm (*p* < 0.001), the number of dead standing trees (*p* < 0.001), the number of trees with liana (*p* < 0.001), and the number of trees with DBH between 30 cm and 50 cm (*p* = 0.002; Figure 7). The number of trees with DBH between 5 cm and 30 cm and proximity to human settlement had no significant effect on the number of omnivore detection.

### 3.7 Spatial autocorrelation

We investigated the spatial distribution of residuals. The spatial distribution of residuals was the result of random spatial process (contiguous forest, Moran's Index = −0.022; *z*‐score = 0.095; *p* = 0.924; Patch 1, Moran's Index = 0.100; *z*‐score = 1.130; *p* = 0.258; Patch 2, Moran's Index = −0.001; *z*‐score = 0.285; *p* = 0.775; Patch 3, Moran's Index = −0.117; *z*‐score = −0.614; *p* = 0.539).

## 4 DISCUSSION

### 4.1 Species composition of forest mammals

Herbivorous and omnivorous species are more tolerant of logging impacts compared to frugivorous and carnivores (Meijaard & Sheil, 2008). Our study confirmed that omnivorous species were the most common at each study site followed by herbivores. Our study showed that the abundance of *Macaca spp*., *M. nemestrina*, and *M. fascicularis* increased in all the forest patches in comparison with contiguous forest. They are commonly known as generalist feeders which they can thrive in the human‐modified environments (Granados et al., 2016; Gumert, 2011; Malaivijitnond & Hamada, 2008). However, mammals affected by fragmentation are at higher risk of extinction, even after considering the effects of important macroecological predictors, such as body size and geographic range size (Crooks et al., 2017). Our data typified the patterns of mammalian biodiversity in urban forest patches on the west coast of Peninsular Malaysia, which is more developed compared to the east coast of the peninsula. Mammalian biodiversity in the east coast is almost double than in our study area (Magintan, Nor, Ean, Lechner, & Azhar, 2017). Mammalian diversity is in impoverished in forest patches with mammal species richness 50% lower than in contiguous forest. At least, 38 mammal species are expected to have once occurred in all the forest patches (Laidlaw, 2000).

Another omnivorous species, *S. scrofa,* had high abundances in all sites, particularly thriving in small forest patches. Surprisingly, in contiguous forest, the average abundance for *S. scrofa* (1.81 individuals per sampling point) was much lower than the urban forest patches. This might be the result of the presence of various competitors including omnivores and herbivores in the contiguous forest which control the wild boar population (Ickes, 2001; Magintan et al., 2017). The absence of large predators such as *Panthera tigris* and *Panthera pardus* in three forest patches would also have resulted in higher wild boar detections as shown in other studies (Ickes, 2001; Lopucki & Kitowski, 2017; Sasidhran et al., 2016). Yet, in Patch 1, average abundance of *S. scrofa* was lower (2.25 individuals) than the other two smaller fragments. This might be caused by hunting as Patch 1 is more exploited by local community of indigenous people, whereas Patch 2 and Patch 3 are rarely used as hunting ground by the indigenous people. Interestingly, the highest average abundance of *S. scrofa* was found in the smallest remnant; Patch 3 (4.89 individuals). *S. scrofa* has a diverse diet which includes fruits, plants, termites, and even human food wastes. Hence, human food wastes may contribute to its diet in these disturbed urban patches especially during times of food scarcity (e.g., Saito & Koike, 2013; Ballari, Conicet, & Barrios‐Garcia, 2014; Morelle et al., 2014).

Laidlaw (2000) reported that the absence of large carnivores in both virgin jungle reserve and logged area of the SLFR. Similarly, our study found an absence of large carnivores, which along with the presence of fast‐growing pioneer vegetation species may relate to the high abundance of the two ungulate species, *M. muntjak* and *T. kanchil* in the study sites (Ballantyne, Gudes, & Pickering, 2014; Granados et al., 2016; McShea et al., 2009; Meijaard & Sheil, 2008). *T. kanchil* was commonly found in the three urban forest patches, possibly due to its small home range and high tolerance toward habitat modification (Meijaard & Sheil, 2008). The presence of domestic dogs in the urban forest patches, particularly Patch 1 and Patch 3, may threaten the *Tragulus spp*. populations. The absence of natural predators may encourage the dogs to fill in the vacuum (Hughes & Macdonald, 2013). The dogs may find the *Tragulus spp*. were common prey animals, occurring in abundant in the urban forest patches. However, the dogs were detected in very few locations (less than 16% or 19 out of 120 sampling points) in all study areas and unlikely to have significant effect on forest mammals.

Only four species of medium‐sized carnivores were found in our study, *Prionailurus bengalensis*,* H. derbyanus,* and *V. megaspila*. Yet, they were not dominant in their contribution to species composition in any of the study sites except for *M. javanica* in Patch 1. The low abundance and diversity of carnivores are unsurprising as the forest patches are too small for their minimum home ranges (Turner & Corlett, 1996). The large carnivores, *P. tigris* and *P. pardus,* were not detected in contiguous forest during the study, but are found in low abundances across the peninsular.

### 4.2 Comparison of habitats

Although secondary forest has conservation value, it is also a prime destination for hunters and poachers (McShea et al., 2009). Our results indicated that Patch 1 (located in between Patch 2 and Patch 3) had the lowest species richness among the four study sites. The presence of many trails are the result of frequent illegal encroachment from nearby neighborhoods and are likely to increase the exposure rate of mammals toward hunting and road kill (Ballantyne et al., 2014; McKinney, 2008; Newbold et al., 2015; Taylor & Goldingay, 2010). Another reason that might contribute to low species composition is the deterioration in habitat quality in this patch as the long‐term effects of habitat isolation. In addition, Patch 1 has been surrounded by high‐density urban areas for a longer period of time than Patch 2 and Patch 3 which are located toward the edges of the Klang Valley.

Surprisingly, Patch 2 and Patch 3 were more similar in term of their species composition, although they are further apart. In addition, both Patch 2 and Patch 3 were smaller than Patch 1. Because Patch 3 was the smallest fragment, it was likely to be more vulnerable by anthropogenic and microclimatic edge effects compared to Patch 1 and Patch 2. However, this does not reflect the diverse species composition of mammals in Patch 3. This could be associated with lower hunting pressure as the local indigenous people that used to live there were relocated to other areas. In addition, small forest patches may present higher species richness due to a greater availability of niche (Pierre & Kovalenko, 2014). However, the patches may not support higher richness across time. Sizeable patches such as Patch 1 and Patch 2 are desirable for conservation outcomes in the urban matrix because these large patches can improve breeding success of forest mammals (Soga & Koike, 2013). Nevertheless, overhunting is likely to negatively affect mammalian biodiversity in Patch 1. Although Patch 2 is the most isolated from the contiguous forest, *T. indicus* was found there. This means Patch 2 could be an important lowland habitat for large mammals despite lacking connectivity to other patches or contiguous forests. The three urban forest patches in this study are surrounded by housing and commercial areas as well as roads/highways. It is very unlikely that the tapirs can safely cross the urban matrix without being adversely affected by anthropogenic activities.

### 4.3 Key factors affecting the mammal species richness, herbivore, and omnivore

There are a range of factors, related to habitat structure driven by historic utilization of the urban forest patches which are likely to affect species richness. In forest landscapes, more than 99% of the species composition is comprised of herbivores and omnivores. Hence, the amount of food resources, such as the number of trees with DBH less than 5 cm, is crucial for species persistence (McShea et al., 2009). The presence of herbivorous and omnivorous species, for example, *T. kanchil*,* M. nemestrina*,* M. fascicularis*, and *S. srofa* in all study sites indicated that small trees provide suitable foraging sites (Adila et al., 2017; Meijaard & Sheil, 2008). While larger diameter trees (e.g., with DBH greater than 30 cm) are vital in providing hiding places for prey species and favorable foraging sites for omnivorous and frugivorous species (Douglas, Vickery, & Benton, 2009; Fuentes‐Montemayor, Goulson, Cavin, Wallace, & Park, 2013; Schaub et al., 2010). Species which exhibit burrowing behavior for nesting and finding food (e.g., termites, worms, and mushrooms) favor dead standing trees that can also provide nesting locations.

Our study found that mammal species richness was negatively influenced by canopy cover, similar to another study by Adila et al. (2017). Dense vegetation with high abundances of standing trees with DBH 5 cm to 30 cm, fallen trees, liana, and palms are all likely to influence movement and the utilization of these landscapes by large‐sized mammal species such as *S. scrofa*,* T. indicus* and *H. malayanus*.

We found that herbivore detection declined with proximity to human settlement. However, overall species richness and omnivore detection did not change with proximity to human settlement. A high human population and the associated settlements can result in a decrease in herbivore hotspots (Bhola, Ogutu, Said, Piepho, & Olff, 2012), particularly among wide‐ranging mammalian herbivores (Torres, Jaeger, & Alonso, 2016).

Our results could contain bias, as the analysis was not corrected for imperfect detection of mammals (Royle & Nichols, 2003). Detection probabilities can provide better estimates of animal populations and disregarding detection probabilities may cause overconfidence, that is, very small confidence intervals except when p is high. However, Welsh, Lindenmayer, and Donnelly (2013) showed that the calculation of detection probabilities can contain similar bias to disregarding nondetection.

### 4.4 Conservation implications

Urban forest fragmentation has impoverished mammalian biodiversity. In our study area, linking the urban forest patches to each other or to contiguous forest would be difficult due to land scarcity, although strategies such as riparian corridors and wildlife crossings should be investigated. The urban forest patches still retained considerable mammal diversity including the presence of the critically endangered Sunda Pangolin and the endangered Sun Bear and Malayan Tapir. Conserving existing urban forest patches is vital to maintain biodiversity in urban landscapes. Hence, we recommend that government stakeholders protect the remaining urban forest habitat and fauna within. In addition, reintroduction of selected species (e.g., tapir, Sambar deer, pangolin, and porcupine), particularly those that will not be involved in serious human‐wildlife conflicts (e.g., tiger, leopard, and elephant), and the restocking of the wild populations in the urban forest patches should be considered (Corlett, 2016).

## CONFLICT OF INTEREST

The authors declare that they have no conflict of interest.

## AUTHOR CONTRIBUTIONS

S.L.T. and B.A. conceived the ideas; S.L.T., N.K., A.Z., and B.A. acquired funding for data collection; S.L.T. and B.A. analyzed the data; S.L.T., L.D.S., N.K., A.Z., A.M.L., A.A., and B.A. wrote the article.

## DATA ACCESSIBILITY

Data are only made available upon request.

We intend to archive data from the manuscript in Dryad Digital Repository.
