# Supplementary material for: Urban forest fragmentation impoverishes native mammalian biodiversity in the tropics
Source: Ecol Evol. 2018 Dec 4;8(24):12506–21. doi: 10.1002/ece3.4632 (PMC6308867; doi:10.1002/ece3.4632)
Supplement: Supplementary file 1 [file ECE3-8-12506-s001.docx]

**Appendix 1.** Correlation matrix of explanatory variables used for modelling mammal species richness.

| **Explanatory variable** | **Code** | **Correlations** | |  |  |  |  |  |  |  |  |  |
| --- | --- | --- | --- | --- | --- | --- | --- | --- | --- | --- | --- | --- |
| Canopy cover | 1 | 1.000 |  |  |  |  |  |  |  |  |  |  |
| The number of dead fallen trees | 2 | -0.096 | 1.000 |  |  |  |  |  |  |  |  |  |
| The number of dead standing trees | 3 | -0.151 | 0.079 | 1.000 |  |  |  |  |  |  |  |  |
| Altitude (m) | 4 | -0.098 | 0.063 | -0.052 | 1.000 |  |  |  |  |  |  |  |
| The number of trees with liana | 5 | 0.068 | 0.085 | 0.144 | 0.059 | 1.000 |  |  |  |  |  |  |
| The number of palms | 6 | 0.144 | -0.032 | 0.369 | -0.164 | 0.112 | 1.000 |  |  |  |  |  |
| The number of shrubs | 7 | -0.26 | 0.197 | -0.131 | 0.051 | 0.047 | -0.243 | 1.000 |  |  |  |  |
| The number of trees with DBH 5cm-30cm | 8 | 0.227 | -0.006 | 0.024 | -0.328 | 0.047 | 0.139 | -0.165 | 1.000 |  |  |  |
| The number of trees with DBH > 50cm | 9 | -0.161 | 0.107 | 0.193 | 0.176 | 0.091 | -0.063 | 0.234 | -0.267 | 1.000 |  |  |
| The number of trees with DBH < 5cm | 10 | 0.185 | 0.225 | 0.035 | -0.283 | 0.109 | 0.145 | 0.15 | 0.234 | -0.135 | 1.000 |  |
| Proximity to human settlement | 11 | 0.006 | -0.139 | -0.124 | 0.562 | 0.249 | -0.111 | 0.089 | -0.279 | 0.026 | -0.224 | 1.000 |
|  |  | 1 | 2 | 3 | 4 | 5 | 6 | 7 | 8 | 9 | 10 | 11 |

**Appendix 2.** Correlation matrix of explanatory variables used for modelling herbivore occurrence.

| **Explanatory variable** | **Code** | **Correlations** | |  |  |  |  |  |  |  |  |  |
| --- | --- | --- | --- | --- | --- | --- | --- | --- | --- | --- | --- | --- |
| Canopy cover | 1 | 1.000 |  |  |  |  |  |  |  |  |  |  |
| The number of dead fallen trees | 2 | -0.096 | 1.000 |  |  |  |  |  |  |  |  |  |
| The number of dead standing trees | 3 | -0.151 | 0.079 | 1.000 |  |  |  |  |  |  |  |  |
| Altitude (m) | 4 | -0.098 | 0.063 | -0.052 | 1.000 |  |  |  |  |  |  |  |
| The number of trees with liana | 5 | 0.068 | 0.085 | 0.144 | 0.059 | 1.000 |  |  |  |  |  |  |
| The number of palms | 6 | 0.144 | -0.032 | 0.369 | -0.164 | 0.112 | 1.000 |  |  |  |  |  |
| The number of shrubs | 7 | -0.26 | 0.197 | -0.131 | 0.051 | 0.047 | -0.243 | 1.000 |  |  |  |  |
| The number of trees with DBH 5cm-30cm | 8 | 0.227 | -0.006 | 0.024 | -0.328 | 0.047 | 0.139 | -0.165 | 1.000 |  |  |  |
| The number of trees with DBH > 50cm | 9 | -0.161 | 0.107 | 0.193 | 0.176 | 0.091 | -0.063 | 0.234 | -0.267 | 1.000 |  |  |
| The number of trees with DBH < 5cm | 10 | 0.185 | 0.225 | 0.035 | -0.283 | 0.109 | 0.145 | 0.15 | 0.234 | -0.135 | 1.000 |  |
| Proximity to human settlement | 11 | 0.006 | -0.139 | -0.124 | 0.562 | 0.249 | -0.111 | 0.089 | -0.279 | 0.026 | -0.224 | 1.000 |
|  |  | 1 | 2 | 3 | 4 | 5 | 6 | 7 | 8 | 9 | 10 | 11 |

**Appendix 3.** Correlation matrix of explanatory variables used for modelling omnivore occurrence.

| **Parameter** | **Code** | **Correlations** | |  |  |  |  |  |  |  |  |  |
| --- | --- | --- | --- | --- | --- | --- | --- | --- | --- | --- | --- | --- |
| Canopy cover | 1 | 1.000 |  |  |  |  |  |  |  |  |  |  |
| The number of dead fallen trees | 2 | -0.096 | 1.000 |  |  |  |  |  |  |  |  |  |
| The number of dead standing trees | 3 | -0.151 | 0.079 | 1.000 |  |  |  |  |  |  |  |  |
| Altitude (m) | 4 | -0.098 | 0.063 | -0.052 | 1.000 |  |  |  |  |  |  |  |
| The number of trees with liana | 5 | 0.068 | 0.085 | 0.144 | 0.059 | 1.000 |  |  |  |  |  |  |
| The number of palms | 6 | 0.144 | -0.032 | 0.369 | -0.164 | 0.112 | 1.000 |  |  |  |  |  |
| The number of shrubs | 7 | -0.26 | 0.197 | -0.131 | 0.051 | 0.047 | -0.243 | 1.000 |  |  |  |  |
| The number of trees with DBH 30cm-50cm | 8 | -0.038 | -0.157 | 0.02 | -0.117 | 0.118 | 0.064 | 0.081 | 1.000 |  |  |  |
| The number of trees with DBH 5cm-30cm | 9 | 0.227 | -0.006 | 0.024 | -0.328 | 0.047 | 0.139 | -0.165 | -0.053 | 1.000 |  |  |
| The number of trees with DBH < 5cm | 10 | 0.185 | 0.225 | 0.035 | -0.283 | 0.109 | 0.145 | 0.15 | -0.125 | 0.234 | 1.000 |  |
| Proximity to human settlement | 11 | 0.006 | -0.139 | -0.124 | 0.562 | 0.249 | -0.111 | 0.089 | -0.002 | -0.279 | -0.224 | 1.000 |
|  |  | 1 | 2 | 3 | 4 | 5 | 6 | 7 | 8 | 9 | 10 | 11 |
